# A New Medical and Surgical Directory of the United States

**Published:** 1885-07

**Authors:** 


Messrs. R. L. Polk & Company, of this city, announce, as in preparation, a new Medical and Surgical Directory, in which they propose to give the name and address of every physician in the United States; also, the name of the college, whether regular or irregular, from which he or she graduated, and the year of graduation, This is to be supplemented by a list of (the names of) Medical Journals published in the United States, names of all Medical Colleges in our country, State Boards of Health, and the laws of different States governing the practice of medicine, and other information relating to the medical profession, of interest to physicians.

